# Myelomeningocele: need for long-time complex follow-up—an observational study

**DOI:** 10.1186/s13013-019-0177-3

**Published:** 2019-03-08

**Authors:** Thomas Bakketun, Nils Erik Gilhus, Tiina Rekand

**Affiliations:** 1 Department of Clinical Medicine, University of Bergen, Bergen, Norway; 2 Department of Neurology, Haukeland University Hospital, Postbox 1400, 5020 Bergen, Norway; 3 Institute for Clinical Neuroscience and Physiology, The Sahlgrenska Academy, University of Gothenburg, Gothenburg, Sweden

**Keywords:** Meningomyelocele, Disability, Neurological disorders, Rehabilitation, Outcome, Adult

## Abstract

**Background:**

Myelomeningocele (MMC) is a congenital disorder that causes a variety of acute as well as late complications. Numerous health problems in adulthood have been described by the persons with MMC but not studied in clinical setting. This study gives implications for organization of the follow-up in adulthood.

**Objectives:**

To investigate the need for follow-up from different medical specialists as well as the need for organized focused rehabilitation among adults with MMC.

**Methods:**

Retrospective cohort study on adults with MMC including multiple departments in a university hospital in Norway. The number and cause of specialized hospital consultations were recorded for every patient. Correlation between childhood health condition related to MMC and the need for specialized consultations in adulthood as well as correlations between number of consultations and anatomical level of MMC, age, and observation time was performed for the whole group.

**Results:**

In total, 38 patients had 672 consultations related to MMC. The most frequent departments were neurology, neurosurgery, urology, gastroenterology, and orthopedics. Most consultations were planned. Complexity of MMC-related health condition correlated to number of specialist consultations (rho = 0.420, *p* = 0.009). Anatomical level of MMC, age, and length of observation time did not correlate with consultations. Pain and shunt failure were the most common reasons for consultations.

**Conclusions:**

Persons with MMC have a need for continuous, life-long multispecialized follow-up and rehabilitation. This is crucial for optimal function, satisfaction with life, and for long-term survival. Systematic follow-up together with rehabilitation will optimize health service.

## Introduction

During fetal development, the brain and spinal cord are formed by folding and closing of the neural plate. Malformations of the spine due to neural tube defects are divided into *spina bifida aperta* (open) and *spina bifida occulta* (closed) [1]. Spina bifida aperta can be divided into meningocele and myelomeningocele (MMC), depending on whether the sac contains only meninges or also neural tissue. Patients with MMC develop a variety of neurological problems including motor, sensory, and autonomic impairment [2–4]. Cerebral malformations may coexist in children with MMC and lead to cognitive impairment. The most common is hydrocephalus, found in 80–90% of MMC patients [1–3]. These patients need often life-long follow-up with shunt revisions [5]. Other coexisting conditions include Arnold-Chiari II malformation, tethered spinal cord, and syringomyelia [2]. Symptoms will usually be evident from early childhood, but some may also develop later [6–8].

Incidence of neural tube defects varies from 0.2/1000 in Japan to 6.1/1000 in Africa and 6.5 / 1000 in the Middle East [9, 10]. Prevalence of neural tube defects in Norway has been estimated to 0.9/1000 and spina bifida to 0.4/1000 in the period 1999–2011 [11]. Most patients with neural tube defects have MMC [9, 12].

Frequency of MMC changes over time and depends on multiple factors such as low folic acid level in pregnant women and abortion rates [11, 13–18]. Since 1998, intake of 400 μg folate daily has been recommended during pregnancy to prevent neural tube defects in Norway [19]. Genetic factors are important and recurrent MMC in the next pregnancy is increased 20-fold [11, 20–22].

Regular follow-up of MMC patients from birth until age 18 years is common clinical practice in most Western countries. Adults with MMC will usually not experience the same systematic control program.

The aim of this study was to identify and characterize the frequency and type of health problems related to MMC in an adult population. We have in particular evaluated all hospital contacts for a well-defined MMC cohort during a long follow-up period.

## Material and methods

We conducted a retrospective cohort study on patients with MMC. The medical records of all patients older than 18 years with ICD-10 code Q05.0–Q05.9 *(spina bifida)* at the Haukeland University Hospital in the period from January 1, 2000, to January 6, 2014, were examined. Both inpatients and outpatients with MMC were included.

Medical impairments and disabilities from childhood as well as use of aids were recorded. The type of consultation, department where consultation was performed, and cause of contact were recorded. Haukeland University Hospital is the primary hospital for a population of approximately half a million.

Correlations between health conditions in childhood, anatomical level of MMC, patient’s age at the end of the study, observation time, and need for specialized consultations were evaluated using regression analysis (Spearman’s rho) and curve estimation. Curve estimation can demonstrate correlations between multiple events in figure. Significance was set at *p* < 0.05.

## Results

In total, 38 adult patients with MMC were included in the study, 11 men and 27 women. The oldest patient was born in 1958, the youngest in 1995 (mean 1979, median 1983).

The patients were observed for 12–173 months; mean observation time 116.8 months and median 119 months. During the observation period, one patient died and four moved out of the region. The MMC was cervical in 2, thoracal in 13, lumbal in 21, and sacral in 2 cases.

Childhood manifestations are listed in Table 1.

**Table 1:** Childhood clinical manifestations in the MMC cohort (*n* = 38)

	*n* (%)
Bladder dysfunction	33 (86.6)
Hydrocephalus	20 (52.6)
Pain	17 (44.7)
Arnold-Chiari malformation	12 (31.6)
Spasticity	11 (28.9)
Scoliosis	11 (28.9)
Tethered cord	10 (26.3)
Psychological problems	10 (26.3)
Cognitive impairment	6 (15.8)

One patient presented six simultaneous health problems, 3 patients had five, 7 patients four, 8 patients three, 11 patients two, and 8 patients had one recorded health problem known in childhood and listed in Table 1.

Mobility aids were used by most patients (Table 2). Three patients used both wheelchair and crutches and braces, five used both wheelchair and crutches, and one used both crutches and braces.

**Table 2:** Mobility aids used by the MMC cohort (*n* = 38)

Aids	*n* (%)
Wheelchair	27 (71.1)
Crutches	10 (26.3)
Orthoses	7 (18.4)
Multiple aids	9 (23.7)
No aids	6 (15.8)
Unknown	1 (2.6)

Table 3 shows the distribution of 625 consultations at the departments most often involved in diagnostics and treatment of MMC. Among the consultations not listed in Table 3 were 28 contacts at the Department of Pulmonology and 19 contacts at the Department of Plastic Surgery related to the MMC. Another 346 consultations at various departments were not necessarily related to MMC.

**Table 3:** Distribution of MMC-related consultations for the most frequently used hospital departments

Medical contacts	Neurology (%)	Neurosurgery (%)	Gastroenterology (%)	Urology (%)	Orthopedics (%)
Outpatient	122 (70.5)	50 (64.1)	63 (82.9)	192 (78.7)	44 (81.5)
Inpatient	51 (29.5)	28 (35.9)	13 (17.1)	52 (21.3)	10 (18.5)
Total (%)	173 (27.5)	78 (12.4)	76 (12.6)	244 (38.9)	54 (8.6)
Planned (%)	161 (93.1)	58 (73.1)	70 (92.4)	207 (89.8)	50 (92.6)

The great majority of the consultations at all departments were planned. Emergency consultations constituted between 7 and 21% at the various departments (Table 3).

The average number of consultations per patient per year was calculated to be 2.6 with variations between < 1 and 14.

**Fig. 1:**
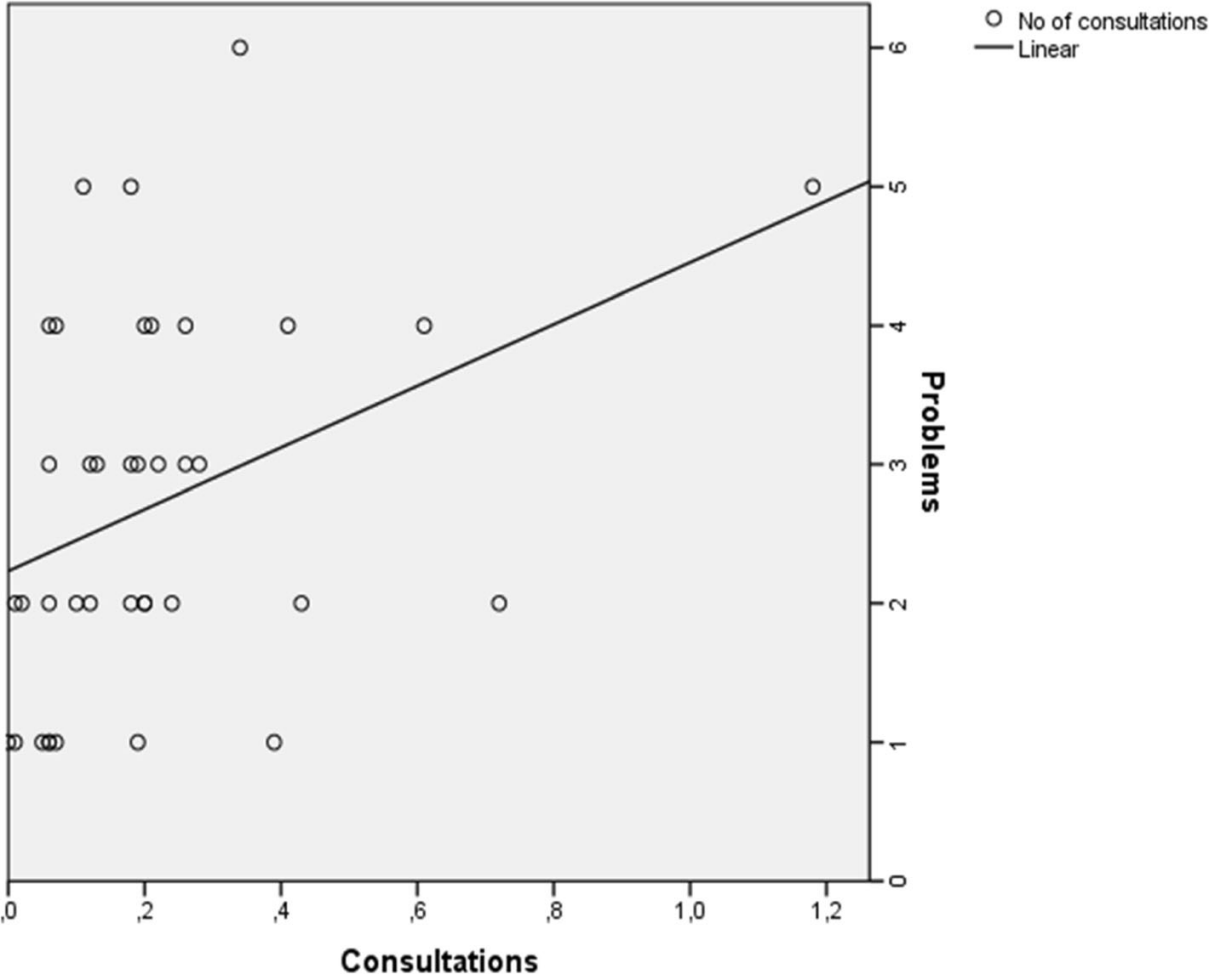
Regression analysis and curve estimation for linear correlation between number of health problems in MMC patients and number of consultations every month

The number of consultations in adulthood correlated to the number of known MMC-related health problems in childhood (Fig. 1; rho = 0.420; *p* = 0.009).

The number of consultations during the observation period did not change. Number of consultations per month and length of observation time were not correlated (rho = 0.032; *p* = 0.859).

There was no correlation between number of consultations and age (rho = 0.01; *p* = 0.907) nor was there any correlation with anatomical level of MMC (rho = 0.062; *p* = 0.276).

We reviewed the causes for hospitalization at Department of Neurology and Department of Neurosurgery in detail (Table 4). Pain and shunt failure were the most common reasons.

**Table 4:** Causes of hospitalizations as inpatients at Department of Neurology and Department of Neurosurgery of adult persons with MMC

Causes	*n* (%)
Pain and headache	19 (50)
Shunt failure	13 (34.2)
Tethered cord	8 (21.1)
Loss of muscle strength	3 (7.9)
Urological and bowel problems	3 (7.9)
Epilepsy	1 (2.6)
Rehabilitation	20 (52.6)
Other	12 (31.6)

## Discussion

We have shown that persons with MMC continue to have a need for clinical follow-up from a wide range of specialists in adulthood. The need for specialized treatments continued unchanged during a long observation period and was related to complexity of health problems known already in childhood. Clinical follow-up did not depend on level of MMC or patients age in our study. Our study revealed that persons with MMC have continuous need for evaluation from different specialties and have need for further follow-up in adult age. Statistical analysis revealed that the number of consultations in adulthood correlated to number of known MMC-related health problems in childhood. Most frequent departments contacted were neurology, neurosurgery, urology, gastroenterology, and orthopedics.

Previous studies demonstrate that adult patients with MMC have an increased mortality rate due to a number of complications [5, 7]. The impact of complications in adulthood on daily life has been reported by persons with spina bifida before [23]. Life-long and specialized follow-up will prevent secondary health problems and further deterioration and should increase quality of life.

Our study demonstrated the need of intervention from different specialists during adulthood, similar to the complex clinical needs described previously during childhood [1]. Consultations from specialists in neurology, neurosurgery, orthopedics, urology, and gastroenterology were continuously needed repeatedly in adult age. Health services used were similar to the needs described for spinal cord injuries [24]. Most consultations were planned. Coordination between specialists increases efficacy of the health services and decreases used time both for health professionals and patients.

No hospital-based psychological or psychiatric evaluations were performed for our adult patients, even though psychologic challenges were described in childhood. Evaluation of cognitive ability may be necessary for advice regarding profession, driving ability and need of help in daily activities. Such aspects may have been overlooked in our patients. Those findings support recommendations given before [25]. Previous studies have shown that despite many challenges, adults with MMC are satisfied with life [26]. However, satisfaction regarding employment and financial independence is low [27]. This supports the need for occupational guidance and if necessary cognitive testing. Our study exposed that some persons with MMC did not meet to planned consultations. Uncoordinated health services combined with cognitive defects in patients probably account for this.

Most neurological dysfunctions related to MMC are already well established in adult age, but new and more debilitating clinical problems can appear. Debilitating pain and a new headache as well as shunt problems because of hydrocephalus can cause a need for neurological intervention with hospitalization. Regular follow-up of the shunt in persons with MMC improves long-term survival [5, 28].

Scoliosis as well as positional and functional failure in the bone-ligament system needs specialized interventions also in adult age as demonstrated in our study. Autonomic dysfunction, particularly from the bladder and bowel, remains to be a challenge for persons with MMC also in adulthood. Our study revealed that patients have a need for both outpatient consultations and hospitalization. Continuous follow-up with urodynamic testing and dietary measures is necessary for life-long optimal functioning [27].

Our study had some limitations, being retrospective and based solely on patient files at the hospital. Problems related to sexuality and pressure ulcers were probably underscored. Previous studies indicate that these are present and influence satisfaction of life [27–31]. Problems with vision and cardiac and endocrinological problems have previously been described but may have been overlooked in our patients [26]. The need for rehabilitation may be underscored as follow-up did not include compulsory evaluation of current functional status and need for aids.

## Conclusions

Our study demonstrates the need for regular and continuous follow-up from several specialists as well as rehabilitation in adulthood. The study implies that such follow-up is crucial for persons with MMC for optimal function, satisfaction with life, and for long-term survival.

## Abbreviation

MMC: Myelomeningocele
